# Palatopharyngeal Arch Staging System (PASS): Consensus about Oropharyngeal Evaluation

**DOI:** 10.3390/life13030709

**Published:** 2023-03-06

**Authors:** Marta Morato, Maribel P. Cardona-Sosa, Gabriela Bosco, Nuria Pérez-Martín, Mayerin M. Marte-Bonilla, Alfonso Marco, Carlos O’Connor-Reina, Rodolfo Lugo, Guillermo Plaza

**Affiliations:** 1 Department of Otolaryngology, Hospital Universitario de Fuenlabrada, Universidad Rey Juan Carlos, 28942 Madrid, Spain; 2 Department of Otolaryngology, Hospital Quirónsalud Madrid, 28223 Madrid, Spain; 3 Ronquido Monterrey, Centro de Diagnóstico y Tratamiento, Monterrey 64660, Mexico; 4 Department of Otolaryngology, Clínica Hospital Constitución, ISSSTE, Monterrey 64530, Mexico; 5 Department of Otolaryngology, Hospital Universitario Sanitas La Zarzuela, 28942 Madrid, Spain; 6 Department of Otolaryngology, Hospital Universitario Reina Sofía, 30003 Murcia, Spain; 7 Department of Otolaryngology, Hospital Quirónsalud Marbella, 29603 Marbella, Spain

**Keywords:** interexaminer agreement, obstructive sleep apnea, palatopharyngeal arch, staging system

## Abstract

Intraoral examinations are essential in the evaluation of the upper airway in patients with obstructive sleep apnea (OSA). The morphology of the anatomic structures of the soft palate, the tonsillar fossae, and the palatoglossus and palatopharyngeal muscles is an important determinant of the size and collapsibility of the velum and oropharynx. The Palatopharyngeal Arch Staging System (PASS) is a systematic way to explore the oropharynx and report anatomic variations in the visible part of the palatopharyngeal muscle. In this prospective study, 30 sleep surgeons evaluated the reliability of the PASS using a selection of 23 videos of oropharyngeal examinations of healthy patients. The corresponding score on the PASS scale was graded for each examination. For internal structure and internal agreement, the Cronbach and Krippendorff alpha values were 0.96 and 0.46, which corresponded to a nearly perfect interrelationship and a moderate agreement, respectively. These findings suggest that the PASS is a valuable tool for evaluating the position of the palatopharyngeus muscle during oropharyngeal examinations and may be useful for creating a common language for sleep surgeons when evaluating the palatopharyngeal muscle.

## 1. Introduction

Obstructive sleep apnea (OSA) is a highly prevalent disease with a complex pathophysiology that entails several anatomic and functional mechanisms [1]. The morphology of the anatomic structures of the soft palate, the tonsillar fossae, and the palatoglossus and palatopharyngeal muscles is an important determinant of the size and collapsibility of the velum and oropharynx. The structure and obstructive pattern of the pharynx differ between people, and the palatopharynx’s muscle position, angulation, and length determine the size and shape of the closure patterns of the retropalatal airway [2]. Therefore, treatment success depends to a large extent on patient selection, which is important for patients undergoing upper airway (UA) surgery [3,4].

The approach to treating OSA is steadily moving from a continuous positive airway pressure-centered “one-size-fits-all” approach to individualized multimodality treatments of UA obstruction [5]. Several staging systems have been proposed for use when planning palatopharyngeal surgeries. Friedman and colleagues correlated oropharyngeal evaluations performed in an office with the prognosis after surgical treatments [3,4]. The current evolution of OSA surgery has moved from simply decreasing the obstruction or enlarging the UA size to stiffening the palate and lateral walls of the oropharynx to minimize the dynamic collapse [5].

The diagnostic approaches and training programs can differ between otolaryngology units and sleep surgeons. Cammaroto et al. [6] reported significant concordance and few divergences of interest in the diagnosis and treatment of sleep disorders between nationalities and types of institutions. Specific scales may be needed to facilitate clear communication between sleep surgeons throughout the world.

Anatomically, the size of the supratonsillar fat pads and the size of the palatine tonsils and their base of implantation in relation to the palatopharyngeus and palatoglossus muscles contribute to individual differences. During presurgical evaluation, it may be helpful to characterize the palatopharyngeus muscle (PPM) because this muscle is involved in most pharyngoplasties. Currently, there is no standardization for its classification as part of physical examinations [7,8].

### Morphological Description of Palatopharyngeus Muscle

The PPM is composed of the aponeurosis and the inferior pterygoid bundles, which originate from a region continuing from the palatine aponeurosis to the inferior margin of the medial pterygoid plate. In the palatine aponeurosis, the palatopharyngeal sphincter, which originates from the nasal aspect of the lateral half of the aponeurosis, passes dorsally below the levator veli palatini (LVP). Therefore, it is distinguishable from the nasal fasciculus, which originates from the medial half of the palatine aponeurosis.

This muscle has the following two major divisions: the longitudinal fascicle and the transverse fascicle. The longitudinal fascicle splits into ventral (small) and dorsal (long) parts, which surround the elevator of the palate velum, before combining into a more concise fascicle that involves the transverse fascicle in the palatopharyngeal arch and the lateral wall of the pharynx. The dorsal and ventral longitudinal heads lie medial to the superior constrictor muscle of the pharynx. Okuda et al. [9] reported that the PPM fascicles comprising the palatopharyngeal arch split into the following two directions: nasal (superficial or luminal layer) and oral (deep layer). The PPM has variations in its form, and these contribute to different anatomic phenotypes, as reviewed by Olszewska and Woodson [2].

**Figure 1:**
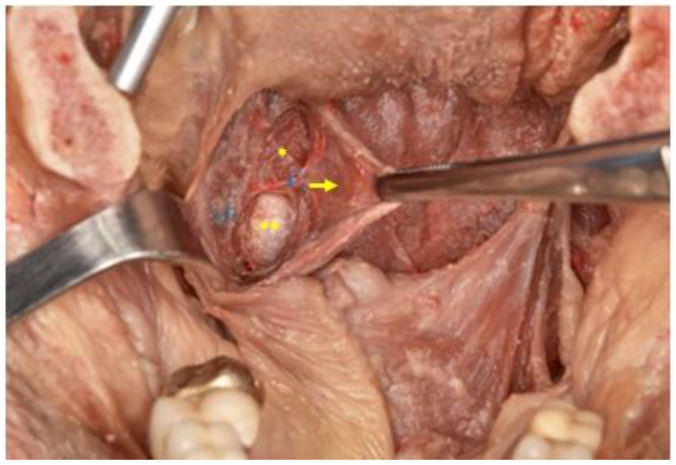
Exposure of the palatopharyngeal muscle (arrow) after medial traction of the posterior pillar and its relationship with the superior constrictor muscle (*) and fascia (**).

These merge from the origin into the posterior part of the palatine aponeurosis and extend in an inferior direction up to its insertion into a large area of the pharyngeal wall (Figure 1).

The most important functions of the PPM include reducing the size of the pharyngeal isthmus, lowering the palate, and raising the larynx. The transition between the PPM and the superior constrictor of the pharynx is known as the palatopharyngeal sphincter.

There are three movements of the walls of the pharyngeal isthmus concerned with the velopharyngeal closure, which are as follows: the backward movement of the anterior wall, the medial movement of the lateral wall, and the forward movement of the posterior wall. Considering the arrangement of muscles around the pharyngeal isthmus (Figure 1), the elevation of the velum by the LVP is responsible for the movement of the anterior wall, while the palatopharyngeal sphincter (PPS) and the superior constrictor of the pharynx (SCP) are responsible for the movement of the lateral and posterior walls. However, this may be ineffective for the movement of the lateral wall owing to its bilateral attachments being fixed in an immovable position by the pterygoid hamulus.

The contraction of the PPM increases the efficiency of the velopharyngeal closure by exerting pressure on the salpingopharyngeal fold and uvula muscles toward the velum. During swallowing, the function of the PPM is constriction through medialization of the lateral wall and shortening of the pharynx. The transverse fascicle flows dorsally from the soft palate to reach the pharyngeal raphe, forming a ridge known as the Passavant’s ridge, through which the soft palate rises to separate the nasopharynx from the oropharynx. Therefore, the PPM acts to raise the pharynx or depress the soft palate and the nasopharyngeal sphincter [8].

However, differences in the position of this muscle in the oropharynx can cause variations in the distance between the contralateral muscle and muscular tone, and these variations contribute to individual differences in anatomic characteristics or functions [10]. Thus, the shape taken by the PPM in its trajectory toward its insertion may help in staging during the preoperative examination of the posterior pillar. The morphology of the fossae palatina and its muscles condition the narrowing of the SPC and contribute to snoring. Lugo et al. [11] designed the Palatopharyngeal Arch Staging System (PASS) as a system for reporting anatomic variations in the PPM during oropharyngeal examinations of patients with OSA. The PASS may be useful for determining the best type of pharyngoplasty for each patient.

The aims of this study were to assess the usefulness of the PASS as a system for reporting anatomic variations in the PPM during oropharyngeal examination and to determine its reliability for standardizing a common language among sleep surgeons.

## 2. Materials and Methods

A prospective, non-randomized, observational, and longitudinal study was designed. Institutional review board approval was obtained from Hospital Universitario de Fuenlabrada, Madrid, Spain (IRB): 22/116.

### 2.1. Population

The participants were healthy patients with adequate sleep hygiene and no complaints of snoring or daytime sleepiness (Epworth Sleepiness Scale < 7 points). We choose to describe and evaluate the distribution of the PPM into the oropharynx only in people without OSA. People with tonsil stages 3 or 4 on the Brodsky scale were excluded from this study because the amount of tonsillar tissue may complicate the PPM examination. Those with Friedman tongue position (FTP) grades 3 or 4 were also excluded because these positions may complicate the morphological assessment of the uvula and PPM.

### 2.2. Staging System

**Figure 2:**
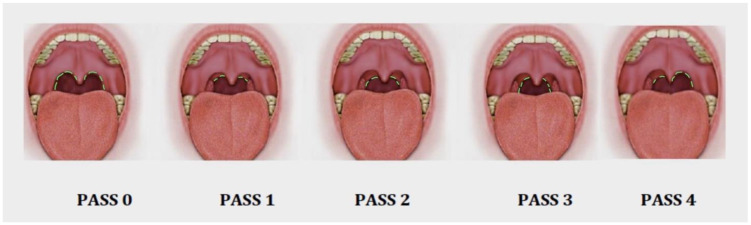
Graphic picture of the PASS. See description in the text.

The PASS [11] comprises a five-item scale that reports on the different possible PPM dispositions during oropharyngeal staging on a scale from 0 to 4 (Figure 2). Videos of each PASS type are included as Supplementary Materials.

The description of each stage is as follows:

- PASS 0: There has been a surgical procedure involving the oropharynx, such as a tonsillectomy or any type of pharyngoplasty. It is not possible to visualize any posterior pillar (PPM).
- PASS 1: The position of the PPM arises from the uvula to the upper pole of both tonsils, and only the upper part of the PPM muscle can be seen. In this stage, most of the PPM is located behind the tonsillar tissue, probably because of a thin PPM.
- PASS 2: The position of the PPM is observed in the upper two-thirds portion from the uvula to the middle pole of the tonsils. A thicker muscle is observed behind the tonsillar tissue, and the upper half of the PPM can be evaluated intraorally.
- PASS 3: The entire portion of the PPM can be observed through the mouth. A thick and powerful muscle is visible behind the tonsillar tissue.
- PASS 4: Asymmetry between the right and left sides is observed when visualizing the PPM.

### 2.3. Digital Video Evaluation

Using a brief description and a graphical representation, the PASS was explained to 30 sleep surgeons from different countries, including Chile, Colombia, the Dominican Republic, Ecuador, Israel, Mexico, Perú, and Spain. This number of reviewers was calculated to provide enough observers to have statistical power in the analysis. These reviewers did not have information about the patients’ clinical history and included members of the network of specialist sleep surgeons from the Ibero-American Society of Sleep Surgery. A total of 23 videos were attached in digital format (five videos are included as Supplementary Materials as Videos S1–S5), each lasting about 15 s, and obtained during office visits by patients at the Hospital Constitución ISSSTE in Monterrey, Nuevo León, Mexico, in May 2021. These videos were sent to the reviewers, who were allowed free access and unlimited time for repeated viewing. This was intended to correspond to different oropharyngeal examinations performed with specific instructions to assess patients with the support of a lingual depressor and the patient saying “A”. The classification was performed using an attached evaluation sheet to determine the scale corresponding to each video. The demographic data, including sex, age, and Brodsky tonsil grade, were collated.

### 2.4. Statistical Analysis

The data were processed using IBM SPSS Statistics (version 26.0; IBM Corp., Armonk, NY, USA). The categorical variables are presented as totals and percentages, and the quantitative variables are presented as means and standard deviations. The intrarater test–retest and interrater reliability were analyzed and used to compare the PASS. The intrarater test–retest reliability covers two related but different concepts: reliability and agreement. Reliability is the ability of a measure applied twice to the same respondents to produce the same ranking on both occasions. Agreement requires the measurement tool to produce the same exact values twice.

An internal structure analysis of the PASS scale was used with the Cronbach α, except for studying correlations between the response patterns, item difficulty, and α for each. A concordance analysis was also performed using the Krippendorff and factorial analyses, which are tests to measure the extent of agreement between raters using multiple categories for classifying the same group of patients. This method can be applied to assess the reliability and reproducibility of diagnostic categorizations of patients and measure the extent of agreement that occurs beyond what would occur by chance alone.

The data factorial analysis was also used to report the main variance components. The results between 0.7 and 0.9 in the validation tests were deemed to be values of interest. For the factorial analysis, *eigen* components were filtered with a value of <0.45, and the colinear results were ruled out.

## 3. Results

**Figure 3:**
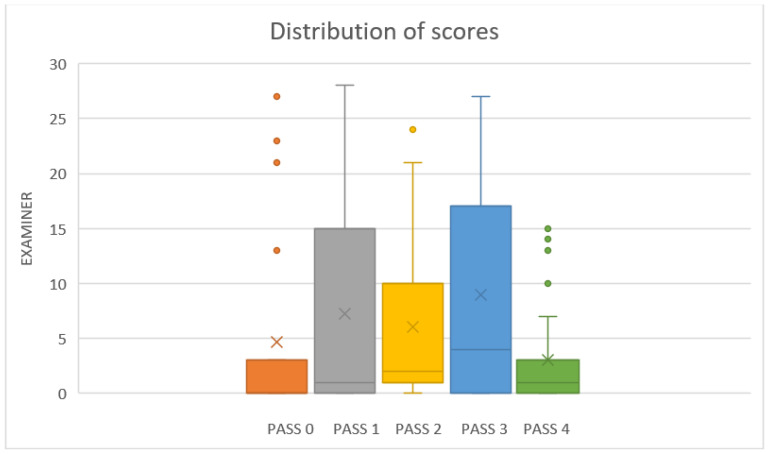
Box-plot distribution of the scores.

The subjects were mainly young adults, with a mean age of 39.7 years (a standard deviation of 9.47), and about 52% (12) of them were women. The tonsils were evaluated as follows: grade 0 was 21.7% (5), grade 1 was 60.8% (14), and grade 2 was 17.5% (4) on the Brodsky scale. Every video was evaluated by 30 sleep surgeons, who used the PASS to score each video. Their results are summarized in Table 1 and Figure 3.

There are five cases in which the SD is more than 1 and different from the other cases. When focusing on these cases, we can see that the confusion factor is the validation of PASS 4, because it induces a wide range of variability when any of the pillars is asymmetric. We can explain these differences in the classification with the pictures in Figure 2, with better accuracy in the rest of the items but not with PASS 4, explaining most of the variability.

**Figure 4:**
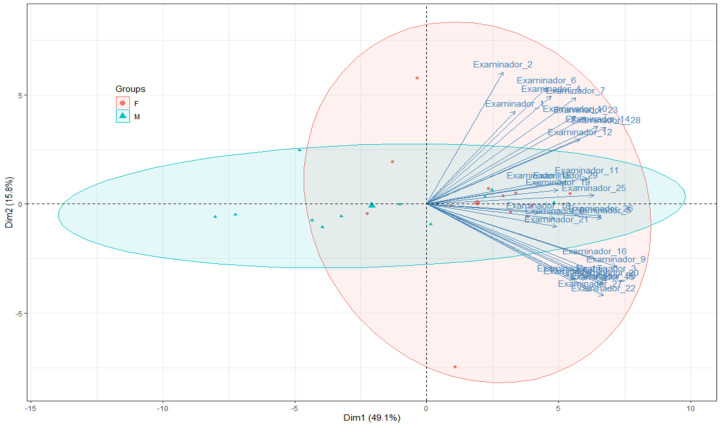
Factorial analysis: distribution of classifications.

The scale’s internal structure was evaluated using the Cronbach α, which showed a high level of internal structure design (α = 0.96) (Table 2). The Krippendorff test was used to evaluate the extent of the agreement, which was 0.46; in a multiple-choice staging system with more than three options, this range of Krippendorff shows a good correlation. As this was a scale, the Kaiser–Meyer–Olkin test for factorial analysis was used to analyze the response pattern (Figure 4). Twenty-eight records were grouped in the first component of variance. Two records were grouped in the second component of variance, which explained only 15% of the variance and showed fewer interrelationships. The two records that were not included in the first component were detected in this second component. The components of variance 3 (11.04%), 4 (6.78%), and 5 (3.89%) together accounted for a very low variance.

The factorial analysis suggested (Figure 4) a high degree of similarity in the response pattern when the 23 records were included in the first component, which accounted for the highest amount of variance in the survey. By the disposition of the response pattern, the two records located in the second component indicated a lack of understanding by two reviewers about how to complete the PASS. This was not detectable in the comparison tests but was found by an analysis of variance.

## 4. Discussion

The PPM is a key anatomic feature in most pharyngoplasties treating the lateral pharyngeal wall [4]. Therefore, a clear description of the PPM is important for sleep surgeons because the morphology, position of the palatal arches, and muscle tone may influence the pattern of palatal obstruction. The aims of this study were to describe the PASS as a system for reporting anatomic variations in the PPM during oropharyngeal examinations and determine its reliability for standardizing a common language among sleep surgeons. In this manuscript, we have shown its use in healthy subjects.

So far, the most common scale used to evaluate the oropharynx is the Friedman tongue position [3,4]. Friedman et al. [4] performed a prospective study of 172 patients who were being evaluated for OSA. They performed a further modified version of the Mallampati examination, where they asked the patients to sit upright with their heads in a neutral position and open their mouths without sticking their tongues out. They initially called this a “modified Mallampati” grade but later changed the term to “Friedman tongue position (FTP)”. They found a statistically significant correlation between the FTP grade and the apnea–hypopnea index (AHI) severity (r = 0.340; *p* < 001). From a clinical perspective, the classification proposed by Friedman et al. is carried out at rest, with the mouth open and the tongue inside the oral cavity. It assesses the tongue distribution with respect to the palate, as well as the tonsil size and BMI. A low Friedman score has been directly related to the possibility of successful surgery following uvulopalatopharyngoplasty (UPPP). However, it does not evaluate the lateral pharyngeal wall.

The main objective of this study was to evaluate the use of the PASS combined with Friedman’s classification to help in the selection of a surgical technique for managing the lateral pharyngeal wall. The combination of the Friedman classification with the PASS is one option because the former evaluates the soft palate and its relationship with the tongue and the latter evaluates the lateral pharyngeal wall, PPM, and tonsil size.

Lateral pharyngeal wall surgery can involve several variations of pharyngoplasty [8,11]. For example, Cahali considered hypertrophy of the PPM as a surgical indication for use of the lateral pharyngoplasty technique [12]. Pang and Woodson [13] based their expansion sphincter pharyngoplasty on the need to modify the PPM. Korhan et al. [14] were among the first to examine the relationship between the oropharyngeal anatomy of the posterior palatal arch at the first examination with the degree of snoring and OSA. They concluded that the distance between the PPM was shorter in patients with severe disease. However, they did not comment on the relationship between these findings and the type of pharyngoplasty.

In 2019, Lugo et al. [11] presented the PASS for evaluating anatomic variations in the PPM during oropharyngeal examinations of patients with OSA. Describing the anatomic variations of the palatopharyngeal arch (PASS 0–4) allows for the evaluation of both the interpalatopharyngeal distance and the PPM tone, which may be helpful when deciding on a specific surgical technique for the lateral pharyngeal wall. For example, for patients with a PASS of 0 or 1, lateral pharyngeal wall mobilization will not be the most important issue, whereas for patients with a PASS of 3 or 4, a specific lateral wall pharyngoplasty would be more appropriate for resolving the collapse.

We believe that it is important to be familiar with the morphology and anatomy of the PPM before deciding on the type of surgery for treating OSA. This is because the management of the oropharynx and its lateral pharyngeal wall is a commonly discussed issue, especially as it relates to the type of collapse. An oropharyngeal office examination may allow clinicians to correlate the features of the PPM with PPM intraoperative findings, with the aim of predicting surgical success or guiding the choice of pharyngoplasty. The broad variety of pharyngoplasty and the differences in terms of PPM mobilization are important when dealing with sleep surgery. Specific lateral pharyngeal wall pharyngoplasty procedures can be performed differently, although all methods share the same goal of lateralizing and stiffening the lateral pharyngeal walls [15].

The PASS classification aims to unify a common terminology among sleep surgeons in terms of intraoral classification of findings in the pharyngeal lateral wall. The present study focused on validating the scale and evaluating the reproducibility of its applications in the office by professionals from different countries and backgrounds. Our intention here was to study the extent of interexaminer agreement, similar to the evaluation of the FTP system published by the Friedman group and other authors [16,17,18,19,20].

Our evaluation shows that the PASS has a consistent internal structure and is well designed. The Cronbach α values were high and similar to those of other classifications, such as the Brodsky scale for tonsil size (k = 0.75) and FTP (k = 0.82) [21,22]. However, our Krippendorff α value was low (0.46). These values show that the concordance between evaluators was not ideal and reflect the heterogeneity among sleep surgeons who did not have complete clinical data to make the evaluation.

Each specialist involved in our study evaluated the cases according to his/her own internal criteria, following the guidelines in the brief explanation given with the PASS scale and the graphic outlines given as a visual support. The surgical training for the treatment of OSA may have differed between these specialists given their professional activities in different parts of the world.

The Krippendorff coefficient found here indicated a lower level of agreement than that reported by other papers on the validation of scales, such as that used to measure lymphatic tissue hypertrophy [16]. Despite this, the Cronbach coefficient in our study indicated a very high internal consistency or reliability (0.96).

The reproducibility of staging systems is an important technical requirement for the scale to have an impact. In addition to the clinical feature it reflects, examining the palatopharyngeal muscle is important in terms of OSA [17].

Although the size of the palatine tonsils, as graded using the Brodsky scale [22], can hinder viewing the posterior pillar during oropharyngeal examinations, the exclusion of patients with hypertrophic tonsils grades 3 or 4 should not affect the validity of the classification. According to the recent Spanish OSA consensus [23], the most appropriate treatment for these patients is a tonsillectomy. For this reason, it is not crucial to classify the type of palatopharyngeal muscle during the physical examination of these patients.

**Figure 5:**
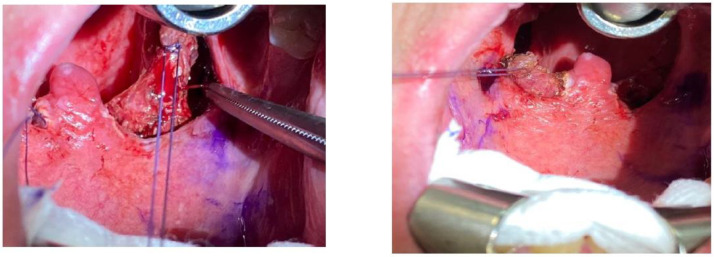
Intraoperative photographs of the palatopharyngeal muscle. In this case, there is a thick palatopharyngeal muscle in both images.

As previously described by Friedman, the relationships between OSAS severity according to the Mallampati score, body mass index, size of the tonsils, and the description of and variations in the PPM are each independently related to the presence and severity of OSAS [24,25]. Given that the PPM is an essential muscle for the surgical treatment of OSA, the proposed PASS may be the first step in identifying the risk or likely outcome of palatine velum surgeries (Figure 5 and Video S6 in Supplementary Materials).

This is even more important when PPM dissection is designed to be performed, either on its own or in combination with some sectioning of the muscle. As some authors are currently promoting, sectioning of the muscle may be indicated in cases with robust PPM, while sparing its anatomy may be an option in cases with thin muscles [7,26]. Thus, studying the PPM prior to or during surgery may be very important to predicting good results.

Our study has some limitations. First, the use of a healthy population allowed for the description of every anatomical possibility without the influence of OSA or any other concurrent phenotype, implying that the evaluation of the PPM was probably modified by other independent factors, such as tongue volume and size, obesity, or nasal obstruction, producing AOS. Our intention is to evaluate this muscle as a self-reliant finding in the oropharynx. Second, the sleep surgeons evaluated the videos but did not examine the patients. This may not have affected the results, but, in some cases, a comprehensive examination of the patient may have been needed. Additionally, as seen in some variations in the interpretation of the videos from the different observers, their different geographical origins may have influenced the results. Third, another limitation is that the external oropharyngeal characteristics of the PPM may be related to the size of the muscle or the number of muscle fibers. This new staging system is intended to be a useful preoperative tool to assess the palatopharyngeal muscle (PPM or posterior pillar). The only way to perform it is during its inspection through the oropharynx during office exams. However, when the tonsils are big, meaning over grade 3, it is impossible to preoperatively assess the PPM, making it best to evaluate it during surgery, especially when repositioning of the muscle is considered.

In recent years, the surgical success rate for treating OSA has increased. This success is related to a better understanding of OSA and the new operative techniques developed specifically and adapted to every patient. All modern pharyngoplasties are concerned with PPM management, which highlights the importance of a better pre-surgical evaluation. Thus, using the PASS may help surgeons determine the best way to treat each OSA patient, highlighting the significance of this study as a way of correlating OSA with PPM anatomy. Further studies are needed to demonstrate the accuracy of the correlation between the PASS and the intrasurgical findings.

## 5. Conclusions

The PASS has a suitable internal design for evaluating the position of the PPM during oropharyngeal examinations. It is an easy scale to learn and put into practice. The evaluation of the oropharyngeal examination videos here shows that the PASS has excellent interrelationships and moderate internal concordance. This scale may be useful as a common language among sleep surgeons for the management of patients with snoring and/or OSA. The use of the PASS to describe the anatomic structures of the lateral pharyngeal wall may be helpful for selecting the appropriate examination and surgical techniques and should be considered for patients with OSA undergoing surgery because of lateral pharyngeal collapse.

## Figures and Tables

**Table 1:** Total PASS score of every video according to the 30 sleep surgeons. SD: standard deviation. * Cases with SD more than 1.

CASES	PASS 0	PASS 1	PASS 2	PASS 3	PASS 4	Median Rate of Score	SD
1	21	0	1	7	1	0.9	1.38 *
2	0	1	4	24	1	2.83	0.51
3	13	1	2	0	14	2.03	1.87 *
4	1	0	2	27	0	2.83	0.57
5	0	14	10	6	0	1.73	0.76
6	3	0	1	26	0	2.67	0.89
7	0	5	21	4	0	1.97	0.54
8	27	3	0	0	0	0.1	0.3
9	0	17	13	0	0	1.43	0.49
10	0	1	13	16	0	2.5	0.55
11	0	0	4	16	10	3.2	0.64
12	2	27	0	0	1	1.03	0.59
13	0	0	17	11	2	2.5	0.61
14	0	21	2	0	7	1.77	1.24 *
15	0	0	1	26	3	3.07	0.35
16	0	0	10	17	2	2.7	0.58
17	2	28	0	0	0	0.93	0.25
18	0	24	6	0	0	1.2	0.39
19	0	1	24	4	1	2.17	0.51
20	13	3	0	1	13	1.93	1.87 *
21	0	15	0	0	15	2.5	1.48 *
22	2	0	7	21	0	2.57	0.79
23	23	5	2	0	0	0.3	0.58

**Table 2:** Interobserver correlation from each examiner.

Examiner	Interobserver Correlation (IC)	Mean of Response	Alpha Cronbach
1	0.397	2000	0.963
2	0.352	2130	0.963
3	0.787	1826	0.960
4	0.564	2043	0.962
5	0.770	2174	0.960
6	0.551	1957	0.962
7	0.679	2087	0.961
8	0.542	1696	0.962
9	0.838	1957	0.960
10	0.682	2174	0.961
11	0.723	1696	0.961
12	0.685	2087	0.961
13	0.764	1739	0.960
14	0.778	1913	0.960
15	0.495	1565	0.963
16	0.735	2000	0.961
17	0.605	1913	0.962
18	0.488	1957	0.962
19	0.588	1913	0.962
20	0.785	2087	0.960
21	0.550	2261	0.962
22	0.770	1696	0.961
23	0.731	2261	0.961
24	0.641	1826	0.961
25	0.750	2000	0.961
26	0.768	2174	0.960
27	0.707	1696	0.961
28	0.820	2043	0.960
29	0.628	1609	0.962
30	0.769	2043	0.960

## Data Availability

Not applicable.

## Supplementary Materials

The following supporting information can be downloaded at: https://www.mdpi.com/article/10.3390/life13030709/s1, Videos S1–S5: Five videos of the 23 patients included in the study, explaining the use of the PASS in these patients, and Video S6: One video showing palatopharyngeal muscle dissection during live surgery.
